# Reliability of Supervised Machine Learning Using Synthetic Data in Health Care: Model to Preserve Privacy for Data Sharing

**DOI:** 10.2196/18910

**Published:** 2020-07-20

**Authors:** Debbie Rankin, Michaela Black, Raymond Bond, Jonathan Wallace, Maurice Mulvenna, Gorka Epelde

**Affiliations:** 1 School of Computing, Engineering and Intelligent Systems Ulster University Derry~Londonderry United Kingdom; 2 School of Computing Ulster University Jordanstown United Kingdom; 3 Vicomtech Foundation Basque Research and Technology Alliance Donostia-San Sebastián Spain; 4 Biodonostia Health Research Institute, eHealth Group Donostia-San Sebastián Spain

**Keywords:** synthetic data, supervised machine learning, data utility, health care, decision support, statistical disclosure control, privacy, open data, stochastic gradient descent, decision tree, k-nearest neighbors, random forest, support vector machine

## Abstract

**Background:**

The exploitation of synthetic data in health care is at an early stage. Synthetic data could unlock the potential within health care datasets that are too sensitive for release. Several synthetic data generators have been developed to date; however, studies evaluating their efficacy and generalizability are scarce.

**Objective:**

This work sets out to understand the difference in performance of supervised machine learning models trained on synthetic data compared with those trained on real data.

**Methods:**

A total of 19 open health datasets were selected for experimental work. Synthetic data were generated using three synthetic data generators that apply classification and regression trees, parametric, and Bayesian network approaches. Real and synthetic data were used (separately) to train five supervised machine learning models: stochastic gradient descent, decision tree, k-nearest neighbors, random forest, and support vector machine. Models were tested only on real data to determine whether a model developed by training on synthetic data can used to accurately classify new, real examples. The impact of statistical disclosure control on model performance was also assessed.

**Results:**

A total of 92% of models trained on synthetic data have lower accuracy than those trained on real data. Tree-based models trained on synthetic data have deviations in accuracy from models trained on real data of 0.177 (18%) to 0.193 (19%), while other models have lower deviations of 0.058 (6%) to 0.072 (7%). The winning classifier when trained and tested on real data versus models trained on synthetic data and tested on real data is the same in 26% (5/19) of cases for classification and regression tree and parametric synthetic data and in 21% (4/19) of cases for Bayesian network-generated synthetic data. Tree-based models perform best with real data and are the winning classifier in 95% (18/19) of cases. This is not the case for models trained on synthetic data. When tree-based models are not considered, the winning classifier for real and synthetic data is matched in 74% (14/19), 53% (10/19), and 68% (13/19) of cases for classification and regression tree, parametric, and Bayesian network synthetic data, respectively. Statistical disclosure control methods did not have a notable impact on data utility.

**Conclusions:**

The results of this study are promising with small decreases in accuracy observed in models trained with synthetic data compared with models trained with real data, where both are tested on real data. Such deviations are expected and manageable. Tree-based classifiers have some sensitivity to synthetic data, and the underlying cause requires further investigation. This study highlights the potential of synthetic data and the need for further evaluation of their robustness. Synthetic data must ensure individual privacy and data utility are preserved in order to instill confidence in health care departments when using such data to inform policy decision-making.

## Introduction

### Background

National health care departments hold volumes of data on patients and the population, and this information is not being used to its full potential due to valid privacy concerns. Machine learning has the potential to improve decisions and outcomes in health care, but these improvements have yet to be fully realized. The reasons may be related to issues facing many data scientists and researchers in this area: the limited availability of or access to data or the readiness of health care institutions to share data. Privacy concerns over personal data, and in particular health care data, means that although the data exist, they are deemed too sensitive for public release [1], even for research purposes.

One way to overcome the issue of data availability is to use fully synthetic data as an alternative to real data. The exploitation of synthetic data in health care is at an early stage and gaining attention. Synthetic data are simulated from real data by using the underlying statistical properties of the real data to produce synthetic datasets that exhibit these same statistical properties. Synthetic data can represent the population in the original data while avoiding any divulgence of real personal, potentially confidential, and sensitive data. In the case of health-related data, this would ensure that actual patient records are not disclosed thus avoiding governance and confidentiality issues. There are three types of synthetic data: fully synthetic, partially synthetic, and hybrid synthetic. This work considers fully synthetic data that does not contain original data.

Synthetic data can be used in two ways: to augment an existing dataset thus increasing its size, for times when a dataset is unbalanced due to the limited occurrence of an event or when more examples are required [2,3] and to generate a fully synthetic dataset that is representative of the original dataset, for times when data are not available due to their sensitive nature [4]. The latter is considered in this work as a key requirement for health care data sharing.

Traditionally, data perturbation techniques such as data swapping, data masking, cell suppression, and adding noise have been applied to real data to modify and thus protect the data from disclosure prior to releasing it. However, such methods do not eliminate disclosure risk and can impact the utility of the data, particularly if multivariate relationships are not considered [5]. Synthetic data was first proposed by Rubin [6] and Little [7]. Raghunathan et al [8] implemented and extended upon this, pioneering the multiple imputation approach to synthetic data generation, exemplified in a range of studies [9-14]. Reiter [15] then introduced an alternative method of synthesizing data through a nonparametric tree–based technique that uses classification and regression trees (CART). A more recent technique proposes a Bayesian network approach for synthetic data generation [16]. Synthetic data is considered a secure approach for enabling public release of sensitive data as it goes beyond traditional deidentification methods by generating a fake dataset that does not contain any of the original, identifiable information from which it was generated, while retaining the valid statistical properties of the real data. Therefore, the risk of reverse engineering or disclosure of a real person is considered to be unlikely [17].

While a number of synthetic data generators have been developed, empirical evidence of their efficacy has not been fully explored. This work extends a preliminary study [18] and investigates whether fully synthetic data can preserve the hidden complex patterns supervised machine learning can uncover from real data and therefore whether it can be used as a valid alternative to real data when developing eHealth apps and health care policy making solutions. This will be achieved by experimenting with a range of open health care datasets. Synthetic data will be generated using three well-known synthetic data generation techniques. Supervised machine learning algorithms will be used to validate the performance of the synthetic datasets. Statistical disclosure control (SDC) methods that can further decrease the disclosure risk associated with synthetic data will also be considered.

### Overview

To inform the viability of the use of synthetic data as a valid and reliable alternative to real data in the health care domain, we will answer the following research questions:

- What is the differential in performance when using synthetic data versus real data for training and testing supervised machine learning models?
- What is the variance of absolute difference of accuracies between machine learning models training on real and synthetic datasets?
- How often does the winning machine learning technique change when training using real data to training using synthetic data?
- What is the impact of SDC (ie, privacy protection) measures on the utility of synthetic data (ie, similarity to real data)?

To answer these questions, 19 open health care datasets containing both categorical and numerical data were selected for experimentation [19]. Synthetic datasets were generated for each dataset using three popular synthetic data generators that apply CART [15,17], parametric [8,17], and Bayesian network [16] approaches to enable a robust comparison of the three synthetic data generation techniques across a broad range of data.

Initially, we analyzed whether the multivariate relationships that exist in the real data were preserved in the synthetic versions of the data for data generated using each of the three synthetic data generation techniques by computing pairwise mutual information scores for each variable pair combination in each dataset [16]. It is important that such relationships are retained when data are synthesized.

To evaluate the utility of synthetic data for machine learning, we then investigated the performance of supervised machine learning models trained on synthetic data and tested on real data compared with models trained on real data and also tested on the real data. This allowed us to determine if a model developed using synthetic data can classify real data examples as accurately and reliably as a model developed using real data. We considered five supervised machine learning models to compare performance and determine if there were differences in robustness across the models. Standard evaluation metrics were computed for models trained on real and synthetic data, for each machine learning model, and for each dataset [20]. The differences in accuracy for models trained on synthetic data versus models trained on real data were computed to analyze the extent to which synthetic data causes a degradation in model performance, if any.

It is pertinent that the optimal machine learning model built using synthetic data matches the optimal machine learning model that would be selected if real data were used in the model training process. This would provide stakeholders in health care with confidence in the use of synthetic data for model development. Thus, we considered how often the best machine learning classifier built using synthetic data matches the best machine learning model built using real data.

Finally, the impact of a number of SDC methods on model performance was assessed. SDC methods seek to further enhance data privacy; however, this can lead to a loss in usefulness of the data [21], and we considered the extent to which performance degradation occurs as a result of SDC.

This large-scale assessment of the reliability of synthetic data when used for supervised machine learning using 19 health care datasets and 3 synthetic data generation techniques provides an important contribution in relation to the trust and confidence that stakeholders in health care can have in synthetic data. We also propose a pipeline to illustrate how synthetic data can potentially fit within the health care provider context. This work demonstrates the promising performance of synthetic data while highlighting its limitations and future work directions to overcome them.

### Synthetic Data: Present and Future Use

The validity and disclosure risk associated with synthetic data has been under investigation by the US Census Bureau since 2003 for the purpose of creating public use data from a combination of sensitive data from the Census Bureau’s Survey of Income and Program Participation, the Internal Revenue Service’s individual lifetime earnings data, and the Social Security Administration’s individual benefit data [22,23]. The goal was to enable the release of synthesized person-level records containing personal and financial characteristics from confidential datasets while preserving privacy. Successful results have led to the release of public use synthetic data files. Researchers can have their work validated against the gold standard (real) data by the Census Bureau, thus enabling them to determine the impact of synthetic data on their exploratory analyses and model development and have confidence in their results while also allowing the Census Bureau to continuously improve their synthesis techniques. The public release of this data has provided significant benefit to the research community and general population, enabling more extensive economic policy research to be performed by groups who could not previously access useful data [24-29]. This work led to the release of further synthetic datasets by the Census Bureau. The Synthetic Longitudinal Business Database comprises data from an annual economic census of establishments in the United States [30]. This dataset provides broad access to rich data that supports the research and policy-making communities in business- and employment-related topics. OnTheMap is a tool using synthetic data to provide information on US citizens such as workforce-related maps, demographic profiles, and reports on analyses of information including the location and characteristics of workers living or working in selected areas, the distance and direction totals between residence and employment locations for workers in selected areas, and disaster event information and the impact of such events on workers and employers [31]. Similarly, synthetic data has also been under investigation in the United Kingdom as a means to provide public access to rich data from UK longitudinal studies [32-34] that contain highly sensitive data linking national census data to administrative data for individuals and their families.

These datasets enable researchers to explore data and develop and test code and models outside the secure environment where real data reside with no restrictions while the data owners provide a mechanism where results, code, and models can be validated on behalf of researchers on the real data within the secure environment and feedback provided. This process increases research productivity while ensuring the development of robust and valid models [35].

While synthetic data have been used to accelerate and democratize business and economic policy research [22-35], the process is not currently in use for health care research, an area that could benefit enormously. With advancements in technology, particularly machine learning and artificial intelligence (AI), the potential to develop diagnostic tools for clinicians and data driven decision-making platforms for health policy-makers is ever increasing [36,37]. Such tools require access to health care data, for example, to train AI algorithms and produce models that can identify health conditions and health-related patterns across the population. Currently, it can take a lengthy period of time for researchers to gain access to health care data, a rich and underused resource, due to privacy concerns [38-42]. For example, in the case of the 40-month Meaningful Integration of Data, Analytics, and Services (MIDAS) Project [36,43] developing a data-driven decision-making tool for health care policy makers, it took more than 20 months to obtain access to the required data due to legal and ethical constraints. In addition, a number of important data variables could not made available, which restricted the utility of the platform under development. With the help of synthetic data, such data, with more or all variables included, could have been made available in a matter of weeks, thus providing more time for development and evaluation of the platform. The platform could then have been installed in health care sites more quickly and connected to real data for validation and comparison of performance for synthetic versus real data, enabling performance tweaks to mitigate bias introduced by synthetic data, if any. Synthetic data could also enable cross-site analytics in various health regions that would enable policy makers to connect their health spaces and potentially provide significant enhancements to cross-national health policy.

The ultimate goal of this work was to further assess the validity and disclosure risk of synthetic data under the stringent conditions associated with health care data with the view to successfully developing a pipeline for use in health care that enables synthetic datasets to be released publicly to researchers, who would otherwise not be able to access the data or access it in a timely fashion, in order to accelerate research by enabling the wider research community to use the data for analysis and model development. The results of such analyses and the models and code developed can then be given to health care departments for validation on the real data and, if effective, put into use by clinicians and health policy-makers.

### Synthetic Data Pipeline for Health Care

**Figure 1:**
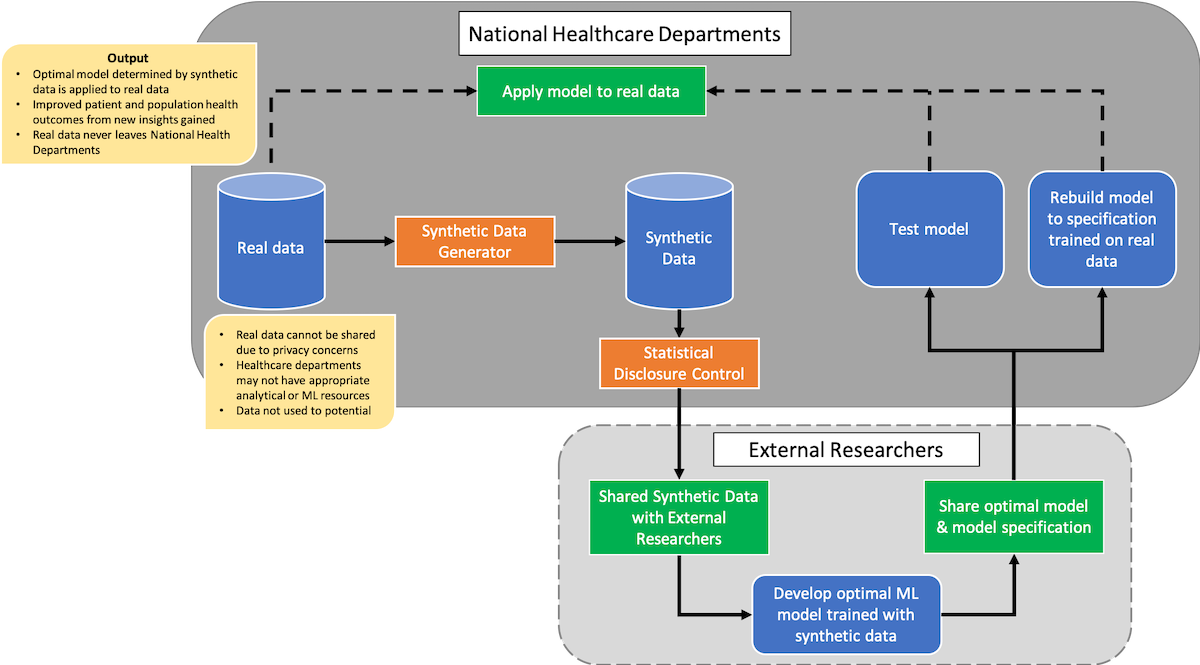
Proposed synthetic data sharing pipeline illustrates how synthetic data could be implemented to expedite health care data analytics.

To understand how health care departments can benefit from synthetic data, we propose the pipeline shown in Figure 1. This is a proposed synthetic data-sharing pipeline provided as an illustration of how synthetic data can potentially work within a real health care setting to expedite data analytics. In future work, we plan to test this pipeline in a real setting. In this pipeline, real data reside within the national health care department infrastructure. The data cannot be shared externally due to the sensitive and private nature. Health care departments may only have a small number of data science staff with the expertise necessary to apply machine learning techniques to many of their datasets, so they cannot maximize the use of their data or discover the potential use of the data due to lack of resources. By applying a synthetic data generation technique to the real data along with SDC measures, a synthetic dataset can be produced and made available to the external research community in place of the real data. External researchers, in large numbers and with wide-ranging expertise, can potentially develop optimal machine learning models trained on the synthetic data and share the performance of the machine learning model, the model itself, and the model specification with the national health care department. The health care department can then test the machine learning model on real data, or in-house technical staff can rebuild the model according to the specification provided by researchers including the program code written by researchers, details of the machine learning algorithm to use (eg, decision tree [DT], support vector machine [SVM]), and the optimal hyperparameter settings determined during development. Using these settings, the model can be rebuilt, this time by training on the real data instead of synthetic data, to which in-house staff have access.

## Methods

### Dataset Selection

For experimentation, 19 open health care datasets have been selected from the University of California Irvine Machine Learning Repository [19]. Missing values have been removed from the datasets either by removing features with a high number of missing values or removing observations where a feature contains a missing value. The experimental datasets and their properties are summarized in Table 1. These datasets were selected to enable an analysis of synthetic data performance when applied to datasets of differing volume and data types (categorical and numerical).

**Table 1:** Summary of experimental datasets.

Dataset and letter designation^a^			Attributes n		Categorical attributes n		Numerical attributes n		Classes/labels n		Observations n
A	Breast Cancer Wisconsin (original)	9		0		9		2		683	
B	Breast Cancer	9		9		0		2		277	
C	Breast Cancer Coimbra	9		0		9		2		116	
D	Breast Tissue	9		0		9		6		106	
E	Chronic Kidney Disease	21		12		9		2		209	
F	Cardiotocography (3 class)	21		0		21		3		2126	
G	Cardiotocography (10 class)	21		0		21		10		2126	
H	Dermatology	34		33		1		6		358	
I	Diabetic Retinopathy	19		3		16		2		1151	
J	Echocardiogram	10		2		8		3		106	
K	EEG^b^ Eye State	14		0		14		2		14980	
L	Heart Disease	13		8		5		2		303	
M	Lymphography	18		18		0		4		148	
N	Postoperative Patient Data	8		8		0		3		87	
O	Primary Tumor	15		15		0		21		336	
P	Stroke	10		7		3		2		29072	
Q	Thoracic Surgery	16		13		3		2		470	
R	Thyroid Disease	22		16		6		28		5786	
S	Thyroid Disease (New)	5		0		5		3		215	
—	Total	283		144		139		105		58,655	

^a^Each dataset has been encoded with a letter (column 1) and will be referenced using this letter for the remainder of the paper.

^b^EEG: electroencephalograph.

### Generating Synthetic Data

In this work, we analyzed and assessed the performance of three publicly available synthetic data generation techniques that are based on well known, seminal work in the area [6-10,15,16]: a parametric data synthesis technique, a nonparametric tree-based synthesis technique that uses CART [15], and a synthesis technique that uses Bayesian networks [16]. While other approaches exist, some are developed for specific datasets and problems (eg, SimPop simulates population survey data [44], and Synthea simulates patient population and electronic health record data [45]), whereas these techniques are considered to be more general. The R package Synthpop, developed by Nowak et al [17], provides a publicly available implementation of the parametric- and CART-based synthetic data generators. The DataSynthesizer python implementation, developed by Ping et al [16], provides a publicly available implementation of the Bayesian network-based synthetic data generator. These implementations have been used in this experimental work.

Attributes were synthesized sequentially in both the parametric and CART methods. The synthetic values for the first attribute were synthesized using a random sample from the original observed data since it has no predictors from previously synthesized attributes in the dataset. When synthesizing attributes, both categorical and numerical, with the nonparametric method, the CART method was applied. CART was applied to all variables that had predictors (ie, attributes prior to them in the sequence) and drew from the conditional distributions fitted to the original data using CART models. The parametric method synthesizes attributes based on data type. Numerical attributes were synthesized using normal linear regression. Categorical attributes were synthesized using polytomous logistic regression where the attribute had more than two levels, and logistic regression was applied to synthesize binary categorical variables [17]. The Bayesian network method of synthesizing data learned a differentially private Bayesian network that captured correlation structure between attributes in the real data and drew samples from this model to produce synthetic data [16].

### Supervised Machine Learning With Real and Synthetic Data

A key measure of data utility of a synthetic dataset for the purpose of machine learning is to determine how well a supervised machine learning model trained on synthetic data performs when tasked with classifying real data. This determines whether supervised machine learning models will be robust enough to classify real data examples if only synthetic data are provided for the training of these models.

To evaluate whether synthetic datasets could be used as a valid alternative to real datasets in machine learning, for each of the 19 datasets (Table 1), five classification models were trained. Initially, the models were trained and tested on the real data to obtain a performance benchmark. Subsequently, a classifier was trained on each of the synthetic datasets, generated using parametric, CART and Bayesian network techniques, and then tested with the real data. Models were tested on real data only to determine whether a model developed by training on synthetic data can be put into use by health care departments and used to accurately classify new, real examples.

The range of models applied to each dataset were stochastic gradient descent DT, k-nearest neighbors (KNN), random forest (RF), and SVM. This selection of algorithms was applied to determine how well each performed when trained with the real data compared with the synthetic data, with both tested on real data.

The classifiers were implemented using Python’s Scikit-Learn 0.21.3 machine learning library and are as follows:

- Stochastic gradient descent classification was implemented using SGDClassifier, a simple linear classifier, with loss=“hinge,” random_state=0 and all other parameters set to their defaults
- DT classification was implemented using DecisionTreeClassifier, an optimized version of CART, with criterion=“gini,” max_depth=10, and random_state=0 and all other parameters set to their defaults
- K-nearest neighbors classification was implemented using KNeighborsClassifier with n_neighbors=10, weights=“uniform,” leaf_size=30, p=2, metric=“minkowski,” n_jobs=2 and all other parameters set to their defaults
- RF classification was implemented using RandomForestClassifier with criterion=“gini,” max_depth=10, min_samples_split=2, n_estimators=10, random_state=1 and all other parameters set to their defaults
- SVM classification was implemented using SVC with C=1.0, degree=3, kernel=“rbf,” probability=True, random_state=None and all other parameters set to their defaults

For training and testing, Python’s Scikit-Learn 0.21.3 ShuffleSplit random permutation cross-validator was used with 10 splitting iterations and a train/test split of 75/25. Categorical attributes were transformed into indicator attributes using one-hot encoding.

### Statistical Disclosure Control

Synthetic data are considered not to contain real units and therefore the risk of disclosure of a real person is considered to be unlikely [46]. While unlikely, the scenario where some of the generated synthetic data are very similar to the real data resulting in potential disclosure risk must be considered, and where additional protections can be applied to synthetic data, it is recommended to do so. Additional SDC measures beyond data synthesis can be applied as a precautionary measure to add further protections to synthetic data by reducing the risk of reproducing real-person records and replicating outlier data, thus further minimizing the risk of disclosure. There are two broad categories of SDC; rules-based SDC consists of a set of fixed rules governing what data can or cannot be released (eg, a rule setting a specific minimum frequency threshold on a dataset in order for it to be released) and principles-based SDC consists of a broader assessment of risk for a dataset to determine whether it is safe for release (eg, in the case where a specific rule on thresholds may not be applicable because the data cannot be linked back to individuals or in cases where thresholds are not enough to protect individuals from reidentification [47]). SDC measures can be applied, evaluated, and reparameterized as part of the penetration and reidentification testing that health care providers would apply before releasing a synthesized dataset.

The following SDC methods, appropriate for rules-based SDC, have been considered and applied in experimental work to determine their effect on data utility:

- Minimum leaf size (CART method specific): for the CART method, a minimum final leaf node size can be set to avoid the risk of final nodes containing small numbers of records, thus increasing the risk of producing real records (and thus real-person data) in the synthesized data. In SDC experiments, this is set to 10.
- Smoothing: smoothing can be applied to continuous/numerical fields in the synthesized data to reduce the risk of releasing unusual/outlier data. In SDC experiments, gaussian kernel density smoothing is applied to numerical attributes only.
- Unique removal: unique records with variable sequences that are identical to records in the real dataset can be removed. In SDC experiments, this has been applied to synthetic data.

Each of these SDC techniques have been applied to the datasets generated using the CART technique, and the smoothing and unique removal techniques have been applied to datasets generated using the parametric technique. SDC methods have not been applied to data synthesized using the Bayesian network technique.

## Results

### Synthetic Data Properties

#### Comparison of Variable Relationships

**Figure 2:**
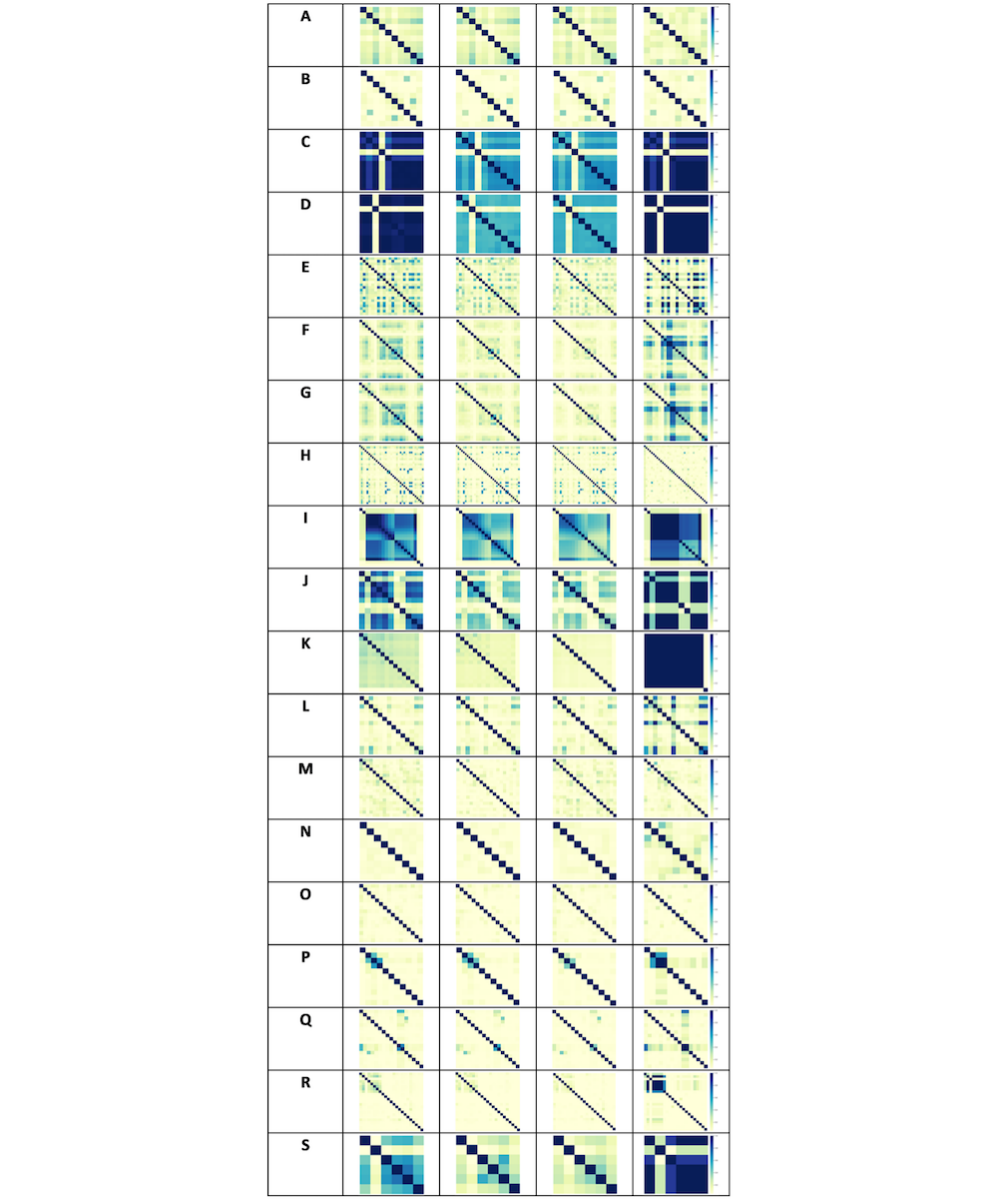
Pairwise mutual information for the real and synthetic datasets. These adjacency heat maps provide an efficient approach to visually determine whether the associations in the real datasets are similar to the associations in the corresponding synthetic datasets. Column 1 indicates the dataset, columns 2 indicates the pairwise mutual information for the real data, and columns 3-5 indicate the pairwise mutual information for synthetic datasets generated using CART, parametric and Bayesian network approaches, respectively.

Within a dataset, relationships can exist between variables. When data are synthesized, we wish to determine whether these relationships are preserved and where they are not preserved, whether this relates to the synthesis technique or structure of the dataset. An analysis of these linear relationships was performed by computing the normalized pairwise mutual information score between each pair of attributes. This is a measure of association or similarity where a higher score indicates a greater association between two attributes. Figure 2 provides a visual representation of the normalized pairwise mutual information scores in adjacency heatmaps for each of the 19 datasets (listed in column 1) and enables visual determination of whether the associations found in the real datasets (column 2) are similar to the associations in the synthetic datasets (columns 3-5) for each of the three synthetic data generators.

The relationships between variables changed slightly in synthetic data generated using the CART and parametric techniques for datasets C-G, I-K, and S, with decreased correlations observed between attribute pairs. These datasets contain mainly and in some cases only numerical attributes. The relationships were largely preserved for the other datasets, which contain mainly and in some cases only categorical attributes, with the exception of dataset A, which contains only numerical attributes.

The relationships between variables also changed slightly in a number of datasets synthesized using the Bayesian network technique (eg, E-G, I-L, N, P-S), with increased correlations observed between attribute pairs. The relationships were largely preserved in datasets B-D, M, and O, while a slight decrease in correlations between attribute pairs was observed for datasets A and H. In this case, the changes cannot be attributed to a particular data type.

### Supervised Machine Learning With Real and Synthetic Data

#### Performance Comparison

To compare the performance of each model when trained on the synthetic data and tested with the real data, a variety of evaluation metrics were used. The accuracy, precision, recall, and F1 score were computed to determine performance.

The accuracy scores for five machine learning models are shown in Table 2 for datasets A through S. Accuracy scores for models trained on the real data and synthetic data are shown where synthetic data is generated using CART, parametric, and Bayesian network techniques, respectively. The accuracy of the models when trained on synthetic data is lower than the accuracy when trained on real data in 92% (263/285) of cases (ie, machine learning results are less accurate for synthetic data in 92% of cases; Table 3).

**Figure 3:**
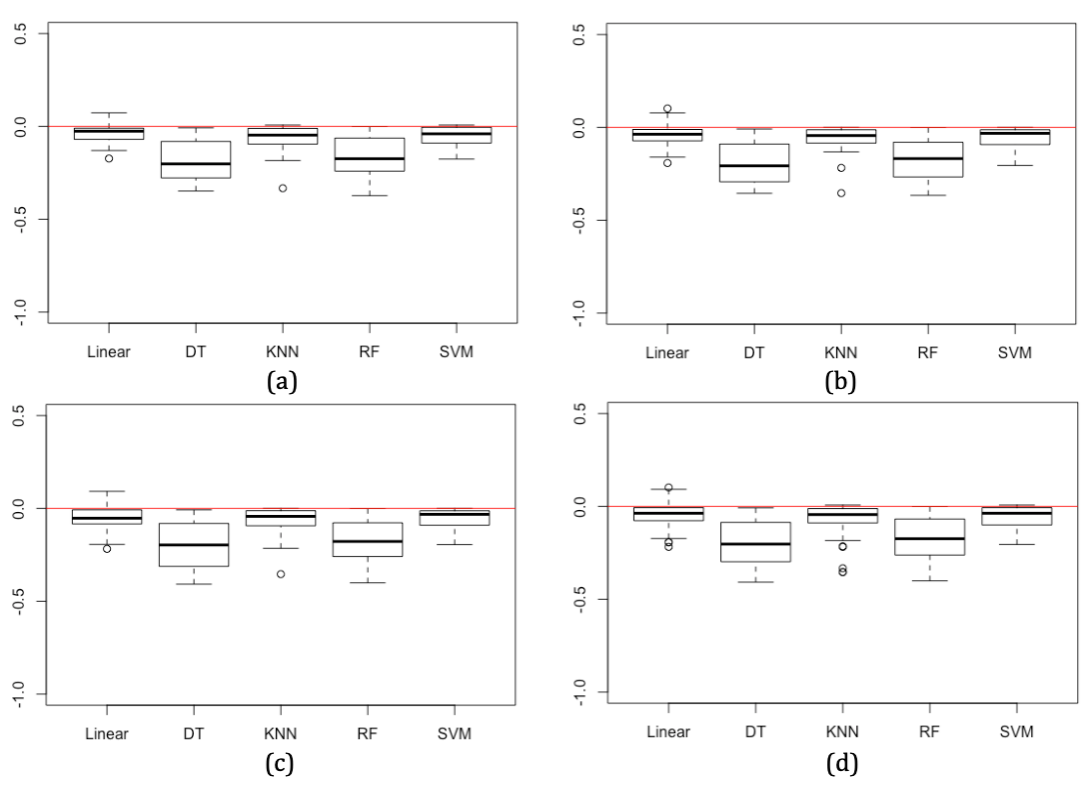
Overall change in accuracy for each machine learning model when trained on synthetic data across 19 datasets and 3 synthetic data approaches where classification and regression tree (a), parametric (b), Bayesian network (c), and all approaches combined (d), compared with models trained using real data.

Although the accuracy decreases in most cases when using synthetic models, this reduction in accuracy is small. The mean absolute difference in accuracy in models trained with synthetic data across all three synthesizing techniques is lowest for SVM, SGD, and KNN models at 0.058 (6%), 0.064 (6%), and 0.072 (7%), respectively. RF and DT models have larger deviations in accuracy at 0.177 (18%) and 0.193 (19%), respectively (Table 4). This pattern is also consistent when considering results for each of the three synthetic data generators separately. These results are illustrated in the boxplots in Figure 3. The mean absolute difference may provide a reliable indicator of the expected decrease in accuracy in supervised machine learning models when developed using synthetic data. A small yet consistent difference in accuracy is expected and manageable between real and synthetic data.

**Figure 4:**
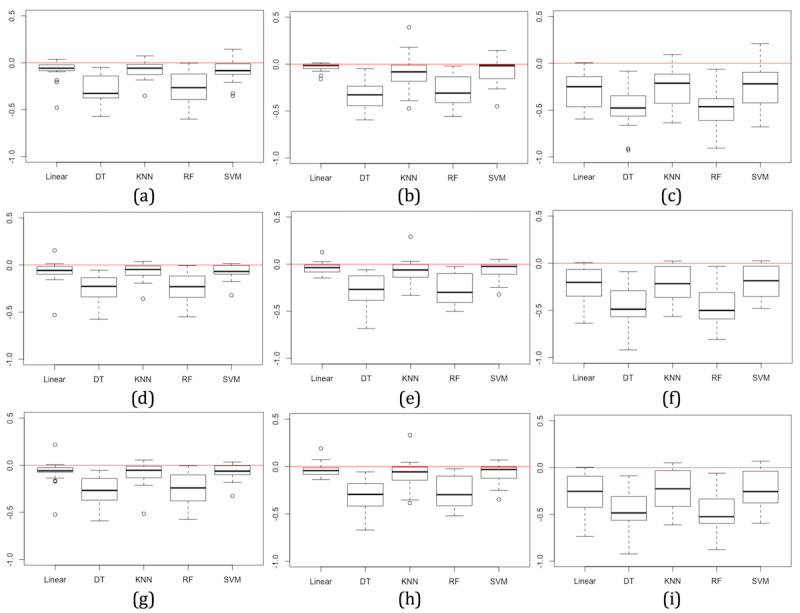
Overall change in precision (a-c), recall (d-f), and F1 (g-i) scores for each machine learning model when trained on synthetic data (from 19 datasets) generated using classification and regression tree (a, d, g), parametric (b, e, h) and Bayesian network (c, f, i) approaches, compared with models trained using real data.

In addition to accuracy scores, we consider changes to precision, recall, and F1 scores. Precision, recall, and F1 scores decrease in almost all models and for data generated with each synthetic data technique across all 19 datasets (Figure 4). These decreases indicate that the models generated with synthetic data have a higher rate of false-positive and false-negative predictions than models trained with real data. Decreases in precision, recall, and F1 are larger in DT and RF models, consistent with changes in accuracy scores; however, the changes are larger than changes in accuracy for these models. The variance in precision, recall, and F1 differences are also more notable in models trained with synthetic data generated using the Bayesian network approach with less problematic decreases observed in models trained with synthetic data generated using the CART and parametric approaches.

**Table 2:** Comparison of accuracy scores of five supervised machine learning models trained on real data and synthetic data across 19 datasets. Increase or decrease in accuracy compared with the model trained on real data shown in parentheses.

Dataset and training set^a^		Machine learning algorithm accuracy				
		SGD^b^	DT^c^	KNN^d^	RF^e^	SVM^f^
**A**						
	Real	0.962	1.000 (W^g^)	0.975	0.997	0.974
	CART^h^	0.966 (+0.004)	0.950 (–0.050)	0.967 (–0.008)	0.965 (–0.032)	0.969 (W) (–0.005)
	Parametric	0.932 (–0.030)	0.907 (–0.093)	0.931 (–0.044)	0.927 (–0.070)	0.946 (W) (–0.028)
	Bayesian	0.954 (–0.011)	0.924 (–0.076)	0.963 (–0.012)	0.947 (–0.050)	0.967 (W) (–0.007)
**B**						
	Real	0.668	0.931 (W)	0.758	0.924	0.83
	CART	0.652 (–0.016)	0.698 (–0.233)	0.765 (+0.007)	0.749 (–0.175)	0.784 (W) (–0.046)
	Parametric	0.706 (+0.048)	0.700 (–0.231)	0.748 (–0.010)	0.726 (–0.198)	0.753 (W) (–0.077)
	Bayesian	0.674 (+0.006)	0.712 (–0.219)	0.744 (–0.014)	0.741 (–0.183)	0.770 (W) (–0.060)
**C**						
	Real	0.629	1.000 (W)	0.784	0.983	0.905
	CART	0.603 (–0.026)	0.652 (–0.348)	0.662 (–0.122)	0.676 (–0.307)	0.729 (W) (–0.176)
	Parametric	0.707 (+0.078)	0.702 (–0.298)	0.652 (–0.132)	0.709 (W) (–0.272)	0.700 (–0.205)
	Bayesian	0.662 (+0.033)	0.709 (–0.291)	0.664 (–0.144)	0.747 (W) (–0.236)	0.710 (–0.195)
**D**						
	Real	0.632	1.000 (W)	0.726	0.962	0.66
	CART	0.502 (–0.130)	0.664 (–0.336)	0.542 (–0.184)	0.706 (W) (–0.254)	0.536 (–0.124)
	Parametric	0.472 (–0.160)	0.666 (W) (–0.334)	0.508 (–0.218)	0.628 (–0.334)	0.545 (–0.115)
	Bayesian	0.438 (–0.194)	0.592 (–0.408)	0.511 (–0.215)	0.649 (W) (–0.313)	0.557 (–0.103)
**E**						
	Real	0.995	1.000 (W)	0.981	1.000 (W)	0.995
	CART	0.972 (–0.023)	0.944 (–0.056)	0.967 (–0.014)	0.995 (W) (–0.005)	0.994 (–0.001)
	Parametric	0.964 (–0.031)	0.981 (–0.019)	0.965 (–0.016)	0.988 (W) (–0.012)	0.988 (W) (–0.007)
	Bayesian	0.986 (–0.009)	0.957 (–0.043)	0.974 (–0.007)	0.992 (–0.008)	0.993 (W) (–0.002)
**F**						
	Real	0.89	0.985 (W)	0.912	0.982	0.913
	CART	0.869 (–0.021)	0.922 (W) (–0.063)	0.883 (–0.029)	0.921 (–0.061)	0.889 (–0.024)
	Parametric	0.873 (–0.017)	0.907 (–0.078)	0.886 (–0.026)	0.914 (W) (–0.068)	0.894 (–0.019)
	Bayesian	0.880 (–0.010)	0.918 (–0.067)	0.885 (–0.027)	0.924 (W) (–0.058)	0.893 (–0.020)
**G**						
	Real	0.746	0.959	0.78	0.971 (W)	0.82
	CART	0.667 (–0.079)	0.848 (W) (–0.111)	0.678 (–0.102)	0.841 (–0.070)	0.748 (–0.072)
	Parametric	0.669 (–0.077)	0.805 (W) (–0.154)	0.676 (–0.104)	0.801 (–0.107)	0.737 (–0.083)
	Bayesian	0.676 (–0.070)	0.835 (W) (–0.124)	0.676 (–0.104)	0.822 (–0.149)	0.739 (–0.081)
**H**						
	Real	1.000 (W)	0.997	0.98	0.994	0.992
	CART	0.940 (–0.060)	0.941 (–0.056)	0.891 (–0.089)	0.958 (W) (–0.036)	0.955 (–0.037)
	Parametric	0.935 (–0.065)	0.951 (–0.046)	0.898 (–0.082)	0.959 (W) (–0.135)	0.959 (W) (–0.032)
	Bayesian	0.940 (–0.060)	0.952 (–0.045)	0.899 (–0.081)	0.955 (–0.139)	0.959 (W) (–0.032)
**I**						
	Real	0.706	0.845	0.711	0.896 (W)	0.676
	CART	0.594 (–0.112)	0.643 (–0.202)	0.634 (–0.077)	0.671 (W) (–0.225)	0.609 (–0.067)
	Parametric	0.570 (–0.136)	0.638 (–0.207)	0.624 (–0.087)	0.663 (W) (–0.233)	0.608 (–0.068)
	Bayesian	0.609 (–0.097)	0.648 (–0.197)	0.629 (–0.082)	0.667 (W) (–0.229)	0.622 (–0.054)
**J**						
	Real	0.453	0.981 (W)	0.642	0.981 (W)	0.651
	CART	0.526 (+0.073)	0.655 (W) (–0.326)	0.579 (–0.063)	0.649 (–0.332)	0.551 (–0.100)
	Parametric	0.555 (+0.102)	0.689 (W) (–0.292)	0.606 (–0.036)	0.628 (–0.354)	0.549 (–0.102)
	Bayesian	0.545 (+0.092)	0.585 (–0.396)	0.585 (–0.057)	0.602 (W) (–0.379)	0.551 (–0.100)
**K**						
	Real	0.551	0.845	0.864	0.885 (W)	0.551
	CART	0.510 (–0.041)	0.531 (W) (–0.314)	0.531 (W) (–0.333)	0.512 (–0.373)	0.531 (W) (–0.020)
	Parametric	0.514 (–0.037)	0.545 (W) (–0.300)	0.510 (–0.354)	0.519 (–0.366)	0.531 (–0.020)
	Bayesian	0.490 (–0.061)	0.538 (W) (–0.307)	0.510 (–0.354)	0.531 (–0.354)	0.510 (–0.041)
**L**						
	Real	0.851	1.000 (W)	0.861	0.977	0.865
	CART	0.791 (–0.060)	0.781 (–0.219)	0.758 (–0.103)	0.803 (W) (–0.174)	0.785 (–0.080)
	Parametric	0.822 (W) (–0.029)	0.758 (–0.242)	0.786 (–0.075)	0.809 (–0.168)	0.793 (–0.072)
	Bayesian	0.785 (–0.066)	0.738 (–0.262)	0.818 (–0.043)	0.799 (–0.178)	0.834 (W) (–0.031)
**M**						
	Real	0.899	1.000 (W)	0.838	0.986	0.939
	CART	0.726 (–0.173)	0.762 (–0.238)	0.762 (–0.076)	0.780 (–0.206)	0.782 (W) (–0.157)
	Parametric	0.739 (–0.160)	0.765 (–0.235)	0.757 (–0.081)	0.772 (–0.214)	0.796 (W) (–0.143)
	Bayesian	0.681 (–0.218)	0.662 (–0.338)	0.703 (–0.135)	0.746 (–0.240)	0.780 (W) (–0.159)
**N**						
	Real	0.713	0.908 (W)	0.713	0.908 (W)	0.713
	CART	0.706 (–0.007)	0.667 (–0.241)	0.715 (+0.002)	0.680 (–0.228)	0.720(W) (+0.007)
	Parametric	0.644 (–0.067)	0.614 (–0.294)	0.706 (–0.007)	0.646 (–0.262)	0.708 (W) (–0.005)
	Bayesian	0.559 (–0.154)	0.591 (–0.317)	0.706 (W) (–0.007)	0.630 (–0.278)	0.694 (–0.019)
**O**						
	Real	0.449	0.732	0.458	0.762 (W)	0.56
	CART	0.338 (–0.111)	0.401 (–0.331)	0.411 (–0.047)	0.410 (–0.352)	0.425 (W) (–0.135)
	Parametric	0.317 (–0.192)	0.377 (–0.355)	0.413 (–0.045)	0.397 (–0.365)	0.433 (W) (–0.127)
	Bayesian	0.293 (–0.156)	0.336 (–0.396)	0.375 (–0.083)	0.361 (–0.401)	0.419 (W) (–0.141)
**P**						
	Real	0.981	0.985 (W)	0.981	0.982	0.981
	CART	0.981 (W) (0.000)	0.977 (–0.008)	0.981 (W) (0.000)	0.981 (W) (–0.001)	0.981 (W) (0.000)
	Parametric	0.981 (W) (0.000)	0.976 (–0.009)	0.981 (W) (0.000)	0.981 (W) (–0.001)	0.981 (W) (0.000)
	Bayesian	0.981 (W) (0.000)	0.977 (–0.008)	0.981 (W) (0.000)	0.981 (W) (–0.001)	0.981 (W) (0.000)
**Q**						
	Real	0.84	0.932 (W)	0.853	0.928	0.851
	CART	0.834 (–0.006)	0.795 (–0.137)	0.850 (–0.003)	0.835 (–0.093)	0.851 (W) (0.000)
	Parametric	0.798 (–0.042)	0.811 (–0.121)	0.848 (–0.005)	0.838 (–0.090)	0.849 (W) (–0.002)
	Bayesian	0.823 (–0.017)	0.794 (–0.138)	0.846 (–0.007)	0.837 (–0.091)	0.851 (W) (0.000)
**R**						
	Real	0.755	0.989 (W)	0.795	0.961	0.738
	CART	0.742 (–0.013)	0.819 (–0.170)	0.761 (–0.034)	0.825 (W) (–0.136)	0.733 (–0.005)
	Parametric	0.749 (–0.006)	0.786 (–0.203)	0.764 (–0.031)	0.798 (W) (–0.163)	0.734 (–0.004)
	Bayesian	0.748 (–0.007)	0.835 (W) (–0.154)	0.762 (–0.033)	0.832 (–0.129)	0.734 (–0.004)
**S**						
	Real	0.958	1.000 (W)	0.921	1.000 (W)	0.953
	CART	0.903 (–0.055)	0.901 (–0.099)	0.899 (–0.022)	0.935 (W) (–0.065)	0.913 (–0.040)
	Parametric	0.890 (–0.068)	0.913 (–0.087)	0.912 (–0.009)	0.930 (W) (–0.060)	0.926 (–0.027)
	Bayesian	0.905 (–0.053)	0.914 (–0.086)	0.908 (–0.013)	0.936 (W) (–0.064)	0.930 (–0.023)

^a^Training dataset name indicates if real or synthetic data were used to train the model and for synthetic datasets which synthetic data generator was used (ie, CART, parametric, or Bayesian).

^b^SGD: stochastic gradient descent.

^c^DT: decision tree.

^d^KNN: k-nearest neighbors.

^e^RF: random forest.

^f^SVM: support vector machine.

^g^(W) highlights the winning classifier for each training set.

^h^CART: classification and regression trees.

**Table 3:** Changes in accuracy for each machine learning model and synthetic data type (19 datasets and 3 synthetic data generators considered providing 57 synthetic datasets to analyze).

Change in accuracy	Machine learning algorithm						
	SGD^a^ (n=57), n (%)	DT^b^ (n=57), n (%)	KNN^c^ (n=57), n (%)	RF^d^ (n=57), n (%)	SVM^e^ (n=57), n (%)		Total (n=285), n (%)
Increase	8 (14)	0 (0)	2 (4)	0 (0)	1 (2)		11 (4)
Same	3 (5)	0 (0)	3 (5)	0 (0)	5 (9)		11 (4)
Decrease	46 (81)	57 (100)	52 (91)	57 (100)	51 (89)		263 (92)

^a^SGD: stochastic gradient descent.

^b^DT: decision tree.

^c^KNN: k-nearest neighbors.

^d^RF: random forest.

^e^SVM: support vector machine.

**Table 4:** Mean absolute difference in accuracy for each machine learning model and synthetic data type.

Synthetic dataset	Mean absolute difference in accuracy per machine learning algorithm				
	SGD^a^, n (%)	DT^b^, n (%)	KNN^c^, n (%)	RF^d^, n (%)	SVM^e^, n (%)
CART^f^	0.053 (5.3)	0.186 (18.6)	0.069 (6.9)	0.164 (16.4)	0.058 (5.8)
Parametric	0.071 (7.1)	0.189 (18.9)	0.072 (7.2)	0.183 (18.3)	0.060 (6.0)
Bayesian network	0.069 (6.9)	0.204 (20.4)	0.075 (7.5)	0.183 (18.3)	0.056 (5.6)
ALL	0.064 (6.4)	0.193 (19.3)	0.072 (7.2)	0.177 (17.7)	0.058 (5.8)

^a^SGD: stochastic gradient descent.

^b^DT: decision tree.

^c^KNN: k-nearest neighbors.

^d^RF: random forest.

^e^SVM: support vector machine.

^f^CART: classification and regression trees.

#### Winning Classifier

In the pipeline described previously, health care departments may wish to release synthetic versions of data to the wider research community for the development of an optimal machine learning model—for example, they may wish to determine the best classifier to use on their real data by making use of the wider range of expertise and scale the external research community can provide. The researchers would be expected to train and test various models and hyperparameters to find the best solution. The researchers would then return a model and/or model specification to the health departments, enabling them to test the model on real data and/or enabling a health department’s technical staff to recreate a version of the model, this time trained on the real data to which in-house staff have access. Health departments would have the expectation that this would be the same model determined if real data had been used to develop the best model (ie, it would have been the “winning” model when trained on either synthetic or real data).

We compared the winning classifier when trained and tested on real data with the winning classifier when trained on synthetic data and tested on real data. Table 2 lists the winning classifier (marked as W on each row) for each dataset when trained with real and synthetic data and when tested on the real data.

The winning classifier when trained on real data matches the winning classifier when trained on synthetic data in only 26% (5/19) of cases for synthetic data generated using the CART and parametric methods, and in just 21% (4/19) of cases on data synthesized using the Bayesian network technique (Table 5).

**Table 5:** Number of instances where the winning classifier trained on synthetic data matches the winning classifier trained on real data across 19 datasets.

Synthetic dataset	Winning classifier matches for real versus synthetic		
	5 classifiers	4 classifiers (DT^a^ removed)	3 classifiers (DT and RF^b^ removed)
CART^c^	5/19 (26.3)	10/19 (52.6)	14/19 (73.7)
Parametric	5/19 (26.3)	10/19 (52.6)	10/19 (52.6)
Bayesian network	4/19 (21.1)	10/19 (52.6)	13/19 (68.4)
All	14/57	30/57	37/57

^a^DT: decision tree.

^b^RF: random forest.

^c^CART: classification and regression trees.

The DT classifier is most often the winning classifier, in 14/19 datasets, when real data are used to train and test the model, but DT is not the best classifier on synthetic data, winning in only 11/57 cases (Table 2). Tree-based methods (DT and RF) are the winning classifier on real data in 18/19 cases (95%). If we remove DTs from this analysis, the cases where the winning classifier when trained on synthetic data matches the winning classifier when trained on real data almost doubles, increasing to 53% (10/19) of cases for synthetic data generated using each of the three synthesizing techniques (Table 5).

With DTs removed, RF models are now the most frequent winners (18/19) when real data are used to train and test the model. In this case, RF models produce the winning classifier in 32/57 cases (Table 2). If we further remove RFs from this analysis and do not consider tree-based classifiers, cases where the winning classifier when trained on synthetic data matches the winning classifier when trained on real data increases from 53% to 74% (14/19) and 68% (13/19) for data synthesized using CART and Bayesian network techniques, respectively, and remains unchanged for data generated using the parametric technique (Table 5).

A chi-square test is applied with the following null and alternative hypotheses:

- H0: the number of winning classifier matches is equal across all sets of classifiers.
- H1: the number of winning classifier matches increases when DT and RF classifiers are removed.

The level of significance adopted for hypothesis testing is .05 for all tests performed.

The null hypothesis is rejected when the tree-based models (DTs and RFs) are removed (ie, from 5 to 3 classifiers) for data synthesized using the CART and Bayesian network methods (Table 6). Therefore, a significant difference in the matching winning classifiers is observed when tree-based classifiers are removed for these two synthesizing techniques. The null hypothesis could not be rejected in all other cases.

**Table 6:** Results of chi-square analysis of the difference in matching winning classifiers for models trained on real versus synthetic data.

Synthetic dataset	Winning classifier matches for real versus synthetic		
	5 classifiers	4 classifiers (DT^a^ removed)	3 classifiers (DT and RF^b^ removed)
CART^c^	0.1843	0.3130	0.0094
Parametric	0.1843	1.0000	0.1843
Bayesian network	0.0927	0.0927	0.0091

^a^DT: decision tree.

^b^RF: random forest.

^c^CART: classification ans regression trees.

#### Impact of Statistical Disclosure Control

The impact of SDC methods on data utility is considered across all datasets. Table 7 illustrates the effect on model accuracy of applying smoothing (numeric attributes only), removal of unique records, and limiting the minimum leaf size (CART models only) to all synthetic datasets where each method is applicable.

**Table 7:** Changes in accuracy for each machine learning model and with statistical disclosure control applied.

Change in accuracy		Machine learning algorithm change in accuracy with SDC^a^					
		SGD^b^	DT^c^	KNN^d^	RF^e^	SVM^f^	Total
**Smoothing (n=150)**							
	Increase	4/30 (13.3)	0/30 (0.0)	1/30 (3.3)	0/30 (0.0)	2/30 (6.7)	7/150 (4.7)
	Same	2/30 (6.7)	0/30 (0.0)	2/30 (6.7)	0/30 (0.0)	3/30 (10.0)	7/150 (4.7)
	Decrease	24/30 (80.0)	30/30 (100.0)	27/30 (90.0)	30/30 (100.0)	25/30 (83.3)	136/150 (90.7)
**Unique removal (n=190)**							
	Increase	4/38 (10.5)	0/38 (0.0)	1/38 (2.6)	0/38 (0.0)	2/38 (5.3)	7/190 (3.7)
	Same	2/38 (5.3)	0/38 (0.0)	2/38 (5.3)	0/38 (0.0)	4/38 (10.5)	8/190 (4.2)
	Decrease	32/38 (84.2)	38/38 (100.0)	35/38 (92.1)	38/38 (100.0)	32/38 (84.2)	175/190 (92.1)
**Minimum leaf size (n=95)**							
	Increase	2/19 (10.5)	0/19 (0.0)	0/19 (0.0)	0/19 (0.0)	1/19 (5.3)	3/95 (3.2)
	Same	1/19 (5.3)	0/19 (0.0)	1/19 (5.3)	0/19 (0.0)	2/19 (10.5)	4/95 (4.2)
	Decrease	16/19 (84.2)	19/19 (100.0)	18/19 (94.7)	19/19 (100.0)	16/19 (84.2)	88/95 (92.6)
**All (n=435)**							
	Increase	10/87 (11.5)	0/87 (0.0)	2/87 (2.3)	0/87 (0.0)	5/87 (5.7)	17/435 (3.9)
	Same	5/87 (5.7)	0/87 (0.0)	5/87 (5.7)	0/87 (0.0)	9/87 (10.3)	19/435 (4.4)
	Decrease	72/87 (82.8)	87/87 (100.0)	80/87 (92.0)	87/87 (100.0)	73/87 (83.9)	399/435 (91.7)

^a^SDC: statistical disclosure control. Each of the 3 types of SDC applied (smoothing, unique removal and minimum leaf size for CART). SDC applied to parametric and CART methods only. Smoothing applied to datasets with numeric attributes only. Minimum leaf size for CART is applicable to CART only.

^b^SGD: stochastic gradient descent.

^c^DT: decision tree.

^d^KNN: k-nearest neighbors.

^e^RF: random forest.

^f^SVM: support vector machine.

In most cases, the machine learning model accuracy decreases when SDC measures are applied to the synthetic data used to train the models. Decreases in accuracy are observed in all DT and RF models and in 83% (72/87), 92% (80/87), and 84% (73/87) of SGD, KNN, and SVM models, respectively. In a small number of cases across SGD, KNN, and SVM models trained on synthetic data with SDC measures applied, no change or a slight increase in accuracy compared with models trained on real data with no SDC measures applied was observed.

**Figure 5:**
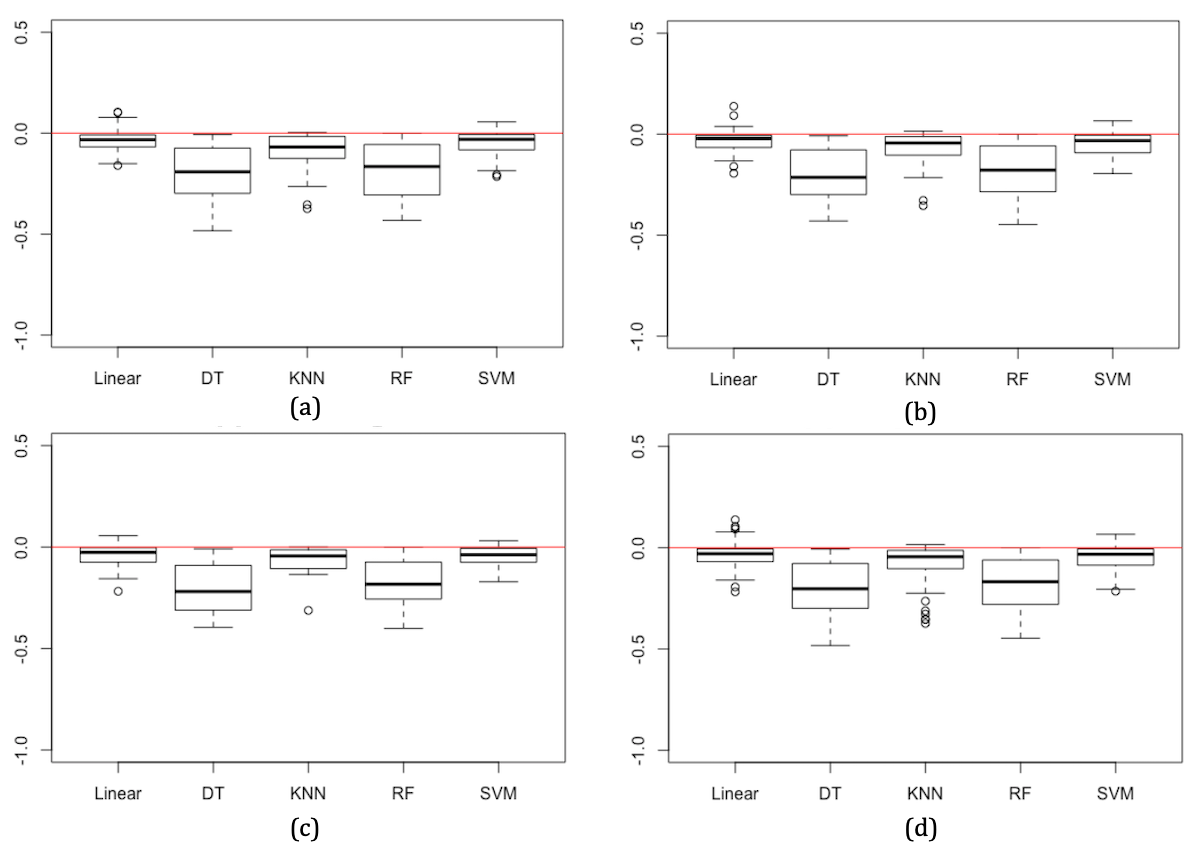
Overall change in accuracy for each machine learning model when trained on synthetic data across 19 datasets and 2 synthetic data approaches (classification and regression tree [CART] and parametric) and with statistical disclosure control measures applied where smoothing (a; numeric attributes only), unique removal (b), minimum leaf size constrained (c; for CART synthesizer only), and all approaches combined (d), compared with models trained using real data.

The mean absolute difference in accuracy when SDC measures are applied to the training data (compared with machine learning models trained on real data) is small across all machine learning models and for all SDC techniques (Table 8). DT and RF models have the largest difference in accuracy, consistent with earlier results of these models trained on synthetic data with no SDC measures applied. The accuracy decreases are consistent across each SDC measure with no SDC measure affecting data utility more notably than any other. These results are also illustrated in the boxplots in Figure 5. Precision, recall, and F1 scores are also consistent with earlier results when no SDC measures are applied. We therefore consider that the SDC techniques investigated do not have a notable impact on data utility beyond what the standard synthesizers have.

**Table 8:** Mean absolute difference in accuracy for each machine learning model and statistical disclosure control type.

SDC^a^ applied to synthetic dataset	Average change in accuracy per machine learning algorithm				
	SGD^b^	DT^c^	KNN^d^	RF^e^	SVM^f^
Smoothing	0.059 (5.9)	0.190 (19.0)	0.094 (9.4)	0.177 (17.7)	0.060 (6.0)
Unique removal	0.052 (5.2)	0.206 (20.6)	0.072 (7.2)	0.184 (18.4)	0.056 (5.6)
Minimum leaf size	0.061 (6.1)	0.200 (20.0)	0.068 (6.8)	0.180 (18.0)	0.053 (5.3)
All	0.056 (5.6)	0.199 (19.9)	0.078 (7.8)	0.180 (18.0)	0.057 (5.7)

^a^SDC: statistical disclosure control.

^b^SGD: stochastic gradient descent.

^c^DT: decision tree.

^d^KNN: k-nearest neighbors.

^e^RF: random forest.

^f^SVM: support vector machine.

We also compare the winning classifier when trained on real data with the winning classifier when trained on synthetic data with SDC applied (Table 9). The winning classifier when trained on synthetic data with SDC applied matches the winning classifier when trained on synthetic data in only 25% (22/87) of cases, consistent with earlier results when SDC measures are not applied. Similar results are observed when each SDC measure is considered individually with the winning classifier matching in models trained with real data compared with models trained using synthetic data with SDC measures of smoothing, unique removal, and minimum leaf size in 27% (8/30), 24% (9/38), and 26% (5/19) of cases, respectively.

Consistent with results in the previous section where SDC measures were not applied, removing tree-based classifiers (DT and RF) from the analysis increases the matches in winning classifiers trained on real compared with synthetic data by 13.3, 36.8, and 36.9 percentage points for each of the SDC measures of smoothing, unique removal, and minimum leaf size, respectively. Overall, an increase of 28.7 percentage points is observed for all SDC measures when tree-based classifiers are removed.

**Table 9:** Number of instances where the winning classifier trained on synthetic data with statistical disclosure control applied matches the winning classifier trained on real data across 19 datasets.

Synthetic dataset	Winning classifier matches for real versus synthetic		
	5 classifiers, n (%)	4 classifiers (DT^a^ removed), n (%)	3 classifiers (DT and RF^b^ removed), n (%)
Smoothing	8/30 (27)	14/30 (47)	12/30 (40)
Unique removal	9/38 (24)	15/38 (40)	23/38 (61)
Minimum leaf size	5/19 (26)	7/19 (37)	12/19 (63)
All	22/87 (25)	36/87 (41)	47/87 (54)

^a^DT: decision tree.

^b^RF: random forest.

A chi-square test is applied with the following null and alternative hypotheses:

- H0: the number of winning classifier matches is equal across all sets of classifiers where SDC measures are applied.
- H1: the number of winning classifier matches increases when DT and RF classifiers are removed where SDC measures are applied.

The level of significance adopted for hypothesis testing is .05 for all tests performed (α=.05).

The null hypothesis is rejected when the tree-based models (DTs and RFs) are removed (ie, from 5 to 3 classifiers) for data synthesized with the SDC measure of unique removal applied (Table 10). Therefore, a significant difference in the matching winning classifiers is observed when tree-based classifiers are removed for this SDC measure. The null hypothesis could not be rejected in all other cases.

**Table 10:** Results of chi-square analysis of the difference in matching winning classifiers for models trained on real versus synthetic data with statistical disclosure control applied.

Synthetic dataset	*P* values		
	5 classifiers	4 classifiers (DT^a^ removed)	3 classifiers (DT and RF^b^ removed)
Smoothing	.18	.79	.41
Unique removal	.22	.11	.003
Minimum leaf size	.73	.19	.05

^a^DT: decision tree.

^b^RF: random forest.

## Discussion

### Principal Findings

The need for synthetic data, particularly in the health care domain, is gaining increasing attention as privacy protection mechanisms are increasingly failing to protect modern data. Due to valid privacy concerns, it is often difficult or impossible to release real health care data thus impeding critical machine learning research that can make use of this data to drive improved patient outcomes and health policy decision-making. Synthetic data has the potential to overcome data availability issues, providing a valid alternative to real data. A small number of synthetic data generators have been proposed in the literature; however, evidence of their efficacy across a large number of datasets and for use in machine learning is thin on the ground.

This work has explored the use of fully synthetic data across 19 health care datasets. Three well-known synthetic data generators have been considered where data is generated using CART, parametric, and Bayesian network techniques. A number of research questions have been answered.

#### What Is the Differential in Performance When Using Synthetic Data Versus Real Data for Training and Testing Supervised Machine Learning Models?

Compared with models trained and tested on real data, almost all machine learning models have a slightly lower accuracy when trained on synthetic data and tested on real data across all synthesizers and for all machine learning models analyzed; however, the average decrease in accuracy was small in all cases. Although still small, DT and RF models had a larger decrease and variance in accuracy than SGD, KNN, and SVM models. In addition to accuracy, an analysis of precision, recall, and F1 scores also showed decreases in scores in models trained with synthetic data, with Bayesian network-generated data resulting in more variance than data generated using CART and parametric techniques.

#### What Is the Variance of Absolute Difference of Accuracies Between Machine Learning Models Training on Real and Synthetic Datasets?

The mean absolute difference was consistently small across all models and synthetic datasets suggesting that these values could provide a reliable indicator of the expected decrease in accuracy in supervised machine learning models when developed using synthetic data. Health care departments could expect a manageable small yet consistent decrease in accuracy between real and synthetic data.

#### How Often Does the Winning Machine Learning Technique Change When Training Using Real Data to Training Using Synthetic Data?

The winning classifier when trained on synthetic data matched the winning classifier when trained on synthetic data in only 26% of cases for synthetic data generated using the CART and parametric methods and in just 21% of cases on data synthesized using the Bayesian network technique across the five machine learning models considered (SGD, DT, KNN, RF, and SVM). Tree-based methods were typically the winning classifier for models trained on real data; however, this was often not the case for models trained on synthetic data. When tree-based models were not considered, the winning classifier when trained on real data matched the winning classifier when trained on synthetic data in 74%, 53%, and 68% of cases for synthetic data generated using the CART, parametric, and Bayesian network approaches, respectively. It would appear that tree-based classifiers have some sensitivity to synthetic data, and the underlying cause requires further investigation.

#### What Is the Impact of Statistical Disclosure Control (ie, Privacy Protection) Measures on the Utility of Synthetic Data (ie, Similarity to Real Data)?

The average change in accuracy when SDC measures are applied to the training data was small across all machine learning models and for all SDC techniques. Again, tree-based models produced the largest decrease in accuracy across all SDC techniques. This is attributed to the synthetic data generation method and not the SDC measures, in line with previous results where SDC measures were not applied. We therefore conclude that the SDC techniques considered do not have a notable impact on data utility beyond what the synthetic data generation methods alone produce.

### Limitations

This work has considered the impact of synthetic data on data utility when the data are used to train supervised machine learning algorithms. Further investigation with a broader range of machine learning algorithms, supervised and unsupervised, and including hyperparameter optimization is required. Such studies should cover an even larger range of datasets including, if possible, real health care department case studies.

Disclosure risk must also be explored in more detail. The impact of SDC measures on data utility has been considered in this work. Disclosure risk must also be measured across synthetic datasets, and a comparison of the data utility and disclosure risk trade-off should be performed.

### Policy and Practice Implications

A wealth of rich health care data exists with the potential to provide new insights for the prevention of diseases, development of personalized medicine, and support of healthy life across the population. These data are held by health care data gatekeepers (eg, national health care departments) and are generally prevented from release, even for research purposes, due to justifiable privacy concerns around the protection of personal data, ethics, and in guaranteeing citizens’ fundamental rights and freedoms.

Data sharing and data use demand careful governance, with legislation such as General Data Protection Regulation and the EU-US Privacy Shield placing increasingly stringent guidelines on data management. Data gatekeepers must manage myriad issues in relation to the nature of the data (eg, categories of sensitive data) and descriptions of the technical characteristics of processed data, as well as sharing and management of the data (eg, fair acquisition, data processing and data retention policies, legal basis for information processing, appropriate security measures) and the configuration of information systems that store and process the data.

From a health care perspective, a range of technical solutions using state-of-the-art machine learning could be developed using health care data with the potential to derive knowledge that can inform and enhance health care policy decision making and risk stratification [36,48]. Such tools can have a positive impact on health policy and practice, meeting the aims of national health departments, for example, as stated by the Department of Health Permanent Secretary in Northern Ireland, Richard Pengelly, in support of the MIDAS project, “the Department seeks to improve the health and social wellbeing of the people of NI, reduce health inequalities, and to assure the provision of appropriate health and social care services in clinical settings and in the community.”

Accessing health care data to develop such tools is complex, involving a lengthy legal and ethical process, and in some cases access is impossible. Synthetic data can potentially overcome the barriers to accessing data and the need for compliance with data protection legislation as they infringe no privacy or confidentiality while remaining durable, reusable, shareable, clean, and potentially reliable as highlighted by Floridi [49], thus accelerating the development of machine learning to inform health care policy. Synthetic data also provide the opportunity to democratize the application of machine learning to health data for the benefit of patients and citizens enabling a larger community to leverage the power of machine learning in health care.

There is an increasing need for the development and evaluation of a robust and trustworthy synthetic data generator. Policy makers and clinicians who base decisions on models developed with synthetic datasets must be able to do so with the assurance that any knowledge elicited is very likely to be reflected in the real data. Using synthetic datasets to facilitate machine learning without disclosing sensitive data has the potential to revolutionize health care research and policy making in an impactful way by unlocking key research data in a secure way that could drive improvements in population health and well-being much more quickly than is currently observed.

### Conclusions

This work considers the efficacy of synthetic data for training supervised machine learning models for use by health care departments. The results are promising with small decreases in accuracy observed in models trained with synthetic data compared with those trained using real data. This work will be further extended to assist in the development of standard baselines for health care departments when using synthetic data (eg, an expected and acceptable decrease in accuracy) and synthetic data generators that can be trusted to produce the same winning model as that which would be produced by real data.

## Abbreviations

AI: artificial intelligence
CART: classification and regression tree
DT: decision tree
KNN: k-nearest neighbors
MIDAS: Meaningful Integration of Data, Analytics, and Services
RF: random forest
SDC: statistical disclosure control
SGD: stochastic gradient descent
SVM: support vector machine
